# Vomiting-induced short gastric artery apoplexy

**DOI:** 10.1259/bjrcr.20150216

**Published:** 2016-09-02

**Authors:** Trishna R Shimpi, Sumer Shikhare, Darren YL Chan, Wilfred CG Peh, Ashish Chawla

**Affiliations:** ^1^Department of Diagnostic Radiology, Khoo Teck Puat Hospital, Alexandra Health, Singapore, Singapore; ^2^Department of General Surgery, Khoo Teck Puat Hospital, Alexandra Health, Singapore, Singapore

## Abstract

Abdominal apoplexy due to short gastric artery rupture following vomiting is an exceedingly rare condition. It results from non-traumatic and non-iatrogenic causes. This entity has variable clinical presentation and patients usually present with non-specific abdominal pain. Imaging plays a vital role in early diagnosis, as immediate exploratory laparotomy is the treatment of choice for successful outcome and helps to reduce mortality rate. We report the case of a 27-year-old male patient who presented to the emergency department with acute-onset abdominal pain after multiple episodes of vomiting following binge alcohol drinking. Contrast-enhanced CT revealed intraperitoneal haemorrhage secondary to vessel rupture, probably from a short gastric artery. Intraoperatively, the short gastric artery was identified as the bleeding source and ligated. The patient had an uneventful postoperative course.

## Clinical presentation and investigations

A 27-year-old male presented to the emergency department with severe epigastric and left hypochondriac pain of 1-day duration. He also complained of pain radiating to the left shoulder, worse with inspiration and lying down. He gave a history of drinking about 1.5 l of alcohol over a period of 2 h. He subsequently had repeated episodes of vomiting, following which he developed severe abdominal pain. The duration, from onset of pain to presentation, was about 8 h. There was no significant past clinical or surgical history. There was no history of abdominal trauma. On physical examination, he was tender, predominantly in the epigastric and left hypochondriac region with no guarding or rebound tenderness. No abdominal distension was noted. The remainder of the physical examination was unremarkable. On presentation, his vital signs were stable; heart rate was 69 min^−1^; blood pressure was 115/77 mmHg; respiratory rate was 18 min^−1^ with alcohol breath positive; temperature was 35.7°C; SpO_2_was 100% with a Glasgow coma scale score of 15/15. He reported a pain score of 10 at presentation (1–10 scale). Laboratory investigations showed a haemoglobin level of 13.7 g dl^−1^ (13–17), leukocyte count of 11.2 × 10^9^ l^−1^ (4–11) and haematocrit of 42.6% (42–52). Prothrombin time, activated partial thromboplastin time and international normalized ratio were within normal limits. Liver function tests and serum amylase level were within normal limits.

## Differential diagnosis

Based on the clinical presentation of several episodes of vomiting followed by acute onset severe abdominal pain after binge alcohol intake, the clinical differentials considered included acute alcoholic pancreatitis and acute gastritis. Other possible differentials considered were Boerhaave’s syndrome and Mallory–Weiss tear, given the multiple episodes of vomiting.

## Imaging findings

**Figure 1.**
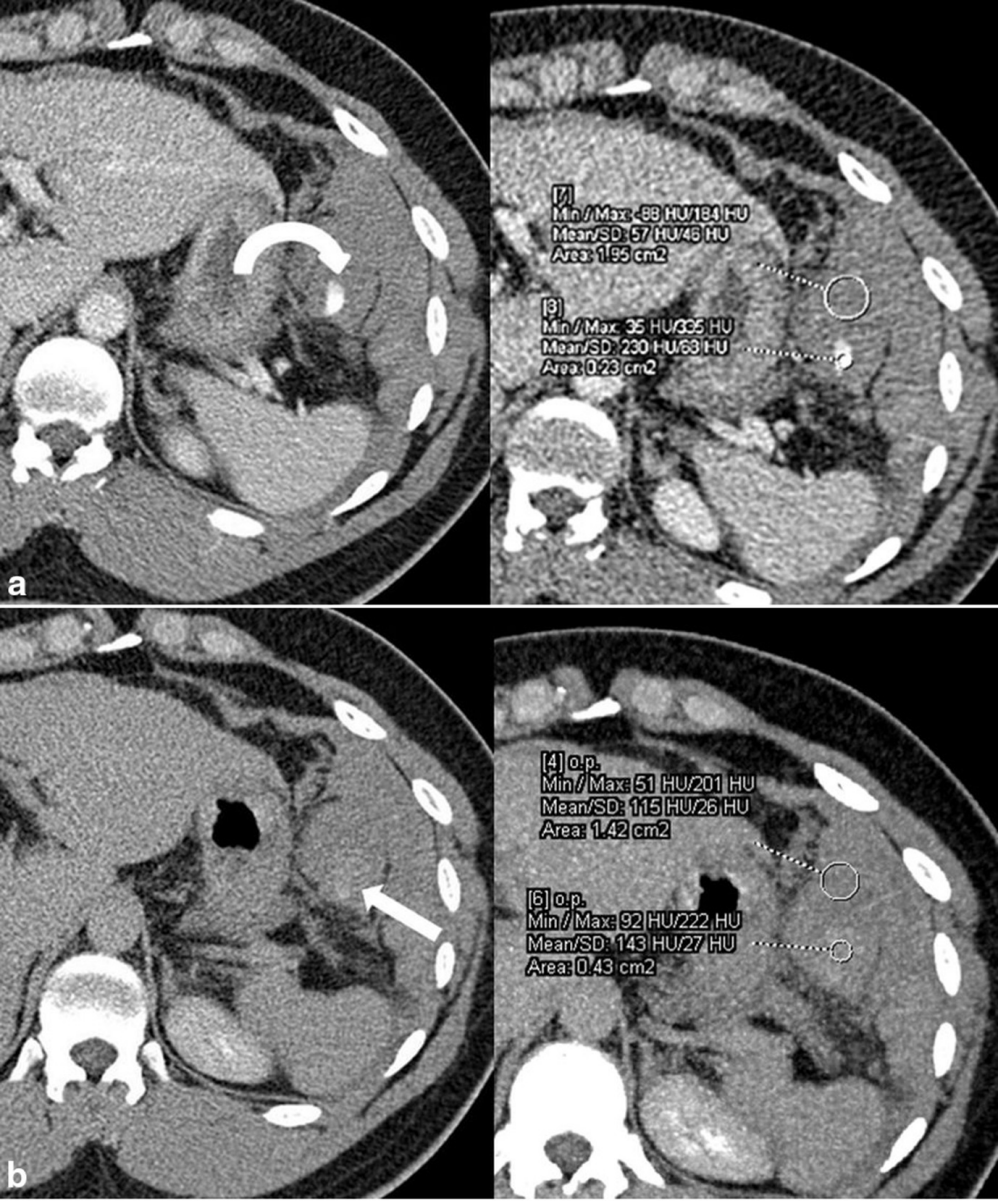
(a) Axial contrast-enhanced CT images of the upper abdomen in portal venous phase show a haematoma in the left hypochondriac region with active contrast extravasation (curved arrow). (b) Axial images in delayed phase show contrast pooling as an area of increased attenuation (arrow) compared with the haematoma.

**Figure 2.**
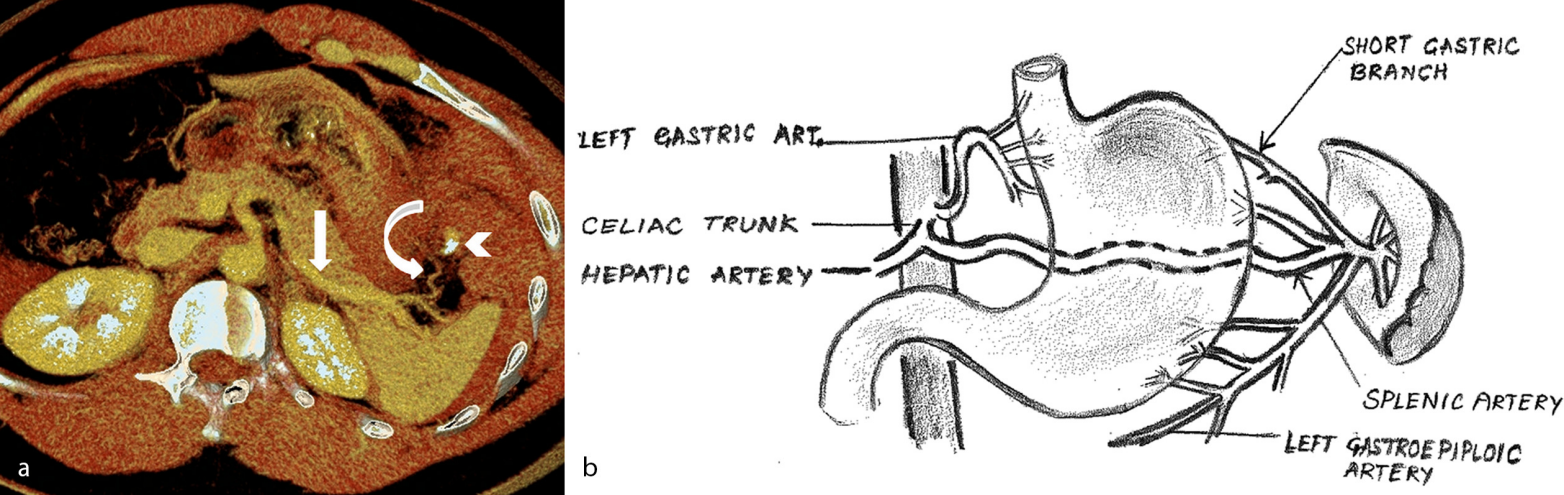
(a) Axial reconstructed maximum intensity projection image shows active contrast extravasation (arrowhead) from the short gastric branch (curved arrow) of the splenic artery (straight arrow). (b) Schematic representation of the splenic artery and its short gastric branches.

Chest and abdominal radiographs were normal. In view of the continuous abdominal pain, contrast-enhanced CT of the abdomen and pelvis was performed. The portal venous phase images demonstrated an approximately 13 × 12 × 10 cm large hyperdense collection [50–60 Hounsfield units (HU)] in the left hypochondriac region adjacent to the greater curvature of the stomach. There was a focus of active contrast extravasation (230 HU) within this collection (Figure 1a). Delayed images revealed contrast pooling within the haematoma, seen as an area of increased attenuation (140–145 HU) compared with the haematoma (110–115 HU) (Figure 1b). The maximum intensity projection images clearly showed contrast leak from smaller branches of the splenic artery (Figure 2a). There was associated haemoperitoneum. Based on the imaging findings, a concern for intraperitoneal haemorrhage secondary to vessel rupture, probably the short gastric artery, was raised (Figure 2b). The findings were immediately conveyed to the referring physician.

## Treatment, outcome and follow-up

**Figure 3.**
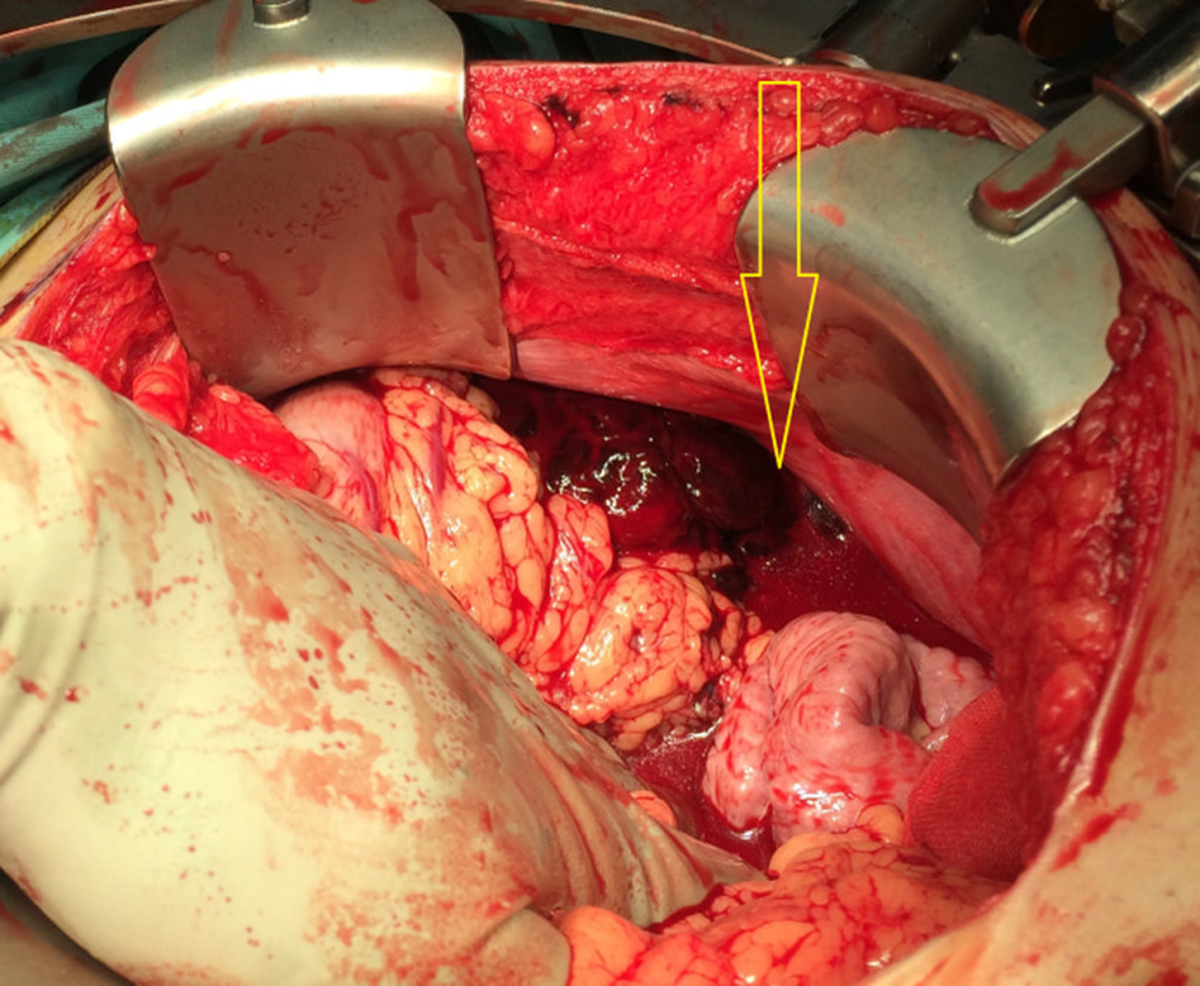
Intraoperative photograph shows haemoperitoneum and blood clots (arrow) along the greater curvature of the stomach.

**Figure 4.**
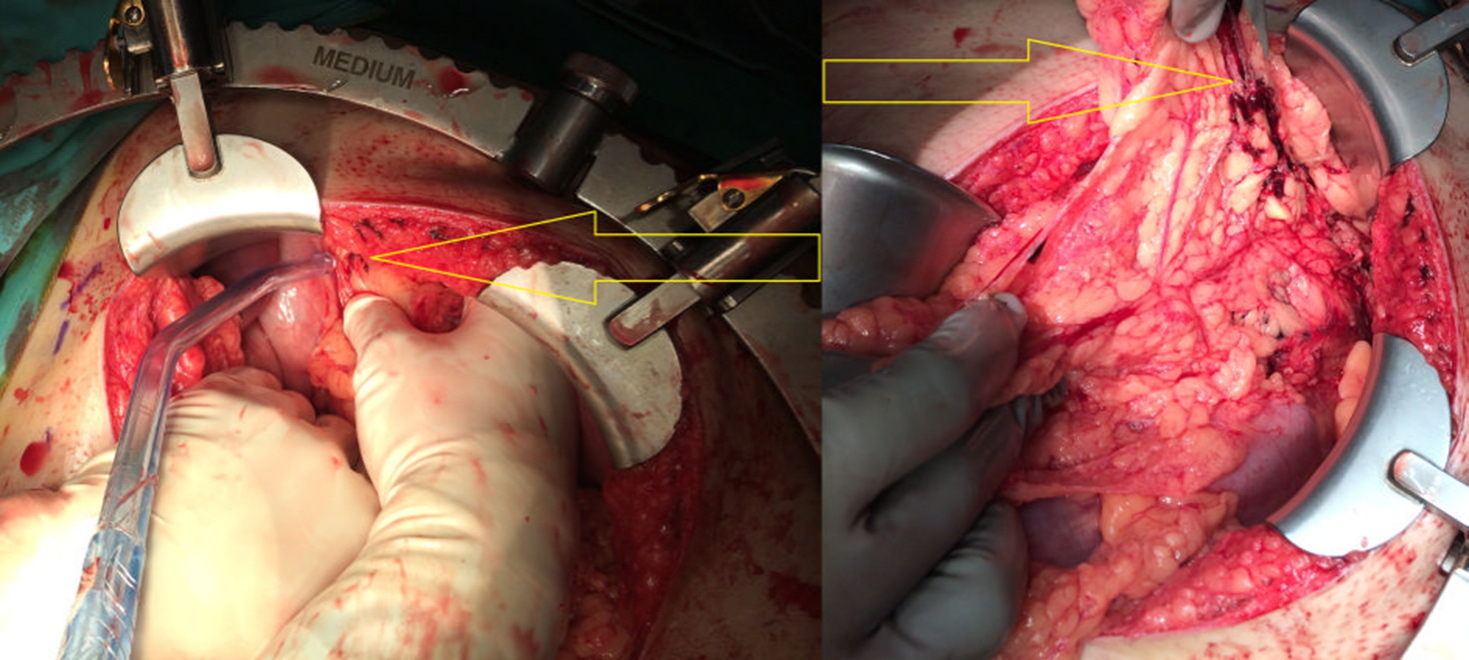
3 Intraoperative photograph showing clots in the area of the short gastric artery, which was identified as the source of bleeding (arrows).

The patient was haemodynamically stable during and after the scan. Repeat haemoglobin and haematocrit levels done 8 h after admission were 11.5 g dl^−1^ (13–17) and 38% (42–52), suggesting volume loss. In view of the imaging findings of active intraperitoneal bleed and falling haemoglobin levels, the patient was immediately taken for exploratory laparotomy. The intraoperative findings echoed the preoperative imaging findings, with the presence of haemoperitoneum. Around 1.2 l of blood clot was evacuated from the peritoneal cavity, largely from around the greater curvature of the stomach (Figure 3). The source of bleeding was found to be the short gastric artery (Figure 4), which was successfully ligated with 3-0 prolene. The stomach was carefully inspected and the gastrocolic ligament and lesser curvature were examined. No bleeding was identified from these sites. No other active source of bleeding was seen. The postoperative recovery was uneventful. Prior to discharge, the patient’s haemoglobin level was 10.6 g dl^−1^ (13–17); hence no blood transfusion was required. The patient was well when discharged 3 days later.

## Discussion

Barber first reported idiopathic spontaneous intraperitoneal haemorrhage in 1909, in a pregnant female with no history of trauma.^1^ The term abdominal apoplexy was coined by Green and Powers^2^ in 1931.^1^ Carmeci et al^3^ found only 110 cases of abdominal apoplexy between 1909 and 1998.^1^ This condition usually occurs in late middle age between 55 and 64 years and affects males more commonly than females in a ratio of 3 : 2.^1^

Most cases of abdominal apoplexy occur secondary to vascular pathology such as arteriosclerosis with or without hypertension; aneurysm of splanchnic artery, mainly splenic and hepatic artery; and vascular erosion caused by an adjacent neoplastic or inflammatory process.^4^ Other causes include malignancy (gastrointestinal stromal tumour, hepatic or renal tumour), gynaecological disorders (ectopic pregnancy rupture, uterine myomas and endometriosis), blunt trauma and idiopathic or excessive anticoagulation.^5^ The common vessels to rupture are the left gastric artery, superior mesenteric artery and the splenic artery. Abdominal apoplexy secondary to rupture of short gastric vessels is exceedingly rare.^1^

It has been postulated that vomiting can predispose to short gastric vessel rupture. Olaoluwakitan et al found only 13 reported cases of short gastric vessel rupture in the literature, of which 8 cases occurred following vomiting.^1^ Hayes et al^6^ postulated that excess vomiting and retching may pull the gastrosplenic ligament, secondary to partial gastric volvulus, creating a shearing force that tears the short gastric artery. In our case, the likely antecedent factor could be vomiting.

Clinically, patients with haemoperitoneum may present in three phases. The initial phase, which is characterized by mild-to-severe abdominal pain due to generalized peritoneal irritation. The pain severity depends on the volume and the rate of bleeding. This is followed by a latent period, which is asymptomatic. In the terminal phase, symptoms usually become more severe and the patient may go into hypovolemic shock.^1^ Our patient presented in the initial phase.

In haemodynamically unstable patients, the diagnosis is generally made on exploratory laparotomy. In such patients, focused assessment by sonography in trauma examination may be a useful preliminary tool to detect intra-abdominal haemorrhage.^7^ In a haemodynamically stable patient, as in our case, contrast-enhanced CT scan of the abdomen and pelvis is the most useful imaging investigation to diagnose abdominal apoplexy preoperatively.^1^ CT scan findings of haemoperitoneum include a sentinel clot, hyperdense mesenteric fluid and active arterial extravasation. These signs help radiologists in identifying the source of intraperitoneal haemorrhage and help direct management.^7,8^ The imaging findings may also help in identifying patients going into hypovolemic shock at an early stage, which may need prompt fluid resuscitation and restoration of circulating volume. The earliest CT imaging sign of decreased vascular volume is flattening of the inferior vena cava. If there is further volume loss leading to hypoperfusion complex, the characteristic imaging findings are increased enhancement of the bowel wall, kidneys, adrenal glands, vasculature and decreased enhancement of the spleen and pancreas. This may help the clinician expedite the initiation of life-saving intervention before the patient’s instability manifests clinically.^9^

Surgery is a definitive treatment for abdominal apoplexy, in addition to restoration of circulating volume. Another treatment option described is transarterial embolization. The choice of treatment is decided based on the age and condition of the patient, as well as the bleeding site.^1^ In haemodynamically stable patients and those who are poor surgical candidates, if the site of active bleeding has been identified on a CT scan, percutaneous transarterial embolization therapy^10,11^ using coils may be considered as a non-invasive, alternative option to surgery. It can also serve as an adjunct to surgery to overcome life-threatening bleeding prior to definitive surgical treatment. However, immediate exploratory laparotomy is required for haemodynamically unstable patients.^11^ Early diagnosis and intervention determines patient prognosis, as non-surgical mortality may reach 100%. Hence, it is imperative to diagnose this entity at an early stage followed by prompt surgical intervention. This may help reduce mortality rates to less than 8.6% if a bleeding point was located and ligated, as opposed to 56% in cases where a bleeding point could not be identified.^1,12^

## Learning points

1. Short gastric artery bleed is a rare cause of abdominal apoplexy, and knowledge of this entity helps in prompt diagnosis and early surgical intervention, escalating the prognosis rate.
2. Abdominal apoplexy secondary to short gastric rupture is known to be associated with frequent bouts of vomiting. Hence, in a patient presenting with abdominal pain following frequent episodes of vomiting, with or without signs of peritonism, the differential of abdominal apoplexy should be kept in mind and investigated promptly.
3. If clinically suspected, early imaging with CT may help achieve a correct preoperative diagnosis of abdominal apoplexy, localize the bleeding site and help direct management.

## Consent

Written informed consent for the case to be published (including images, case history and data) was obtained from the patient for publication of this case report.
